# Two-year clinical outcome of denosumab treatment alone and in combination with teriparatide in Japanese treatment-naive postmenopausal osteoporotic women

**DOI:** 10.1038/boneres.2016.55

**Published:** 2017-06-13

**Authors:** Yukio Nakamura, Takako Suzuki, Mikio Kamimura, Shota Ikegami, Kohei Murakami, Shigeharu Uchiyama, Akira Taguchi, Hiroyuki Kato

**Affiliations:** 1 Department of Orthopedic Surgery, Shinshu University School of Medicine, Matsumoto, Japan; 2 Department of Orthopedic Surgery, Showa-Inan General Hospital, Komagane, Japan; 3 Center of Osteoporosis and Spinal Disorders, Kamimura Orthopaedic Clinic, Matsumoto, Japan; 4 Department of Oral and Maxillofacial Radiology, Matsumoto Dental University, Shiojiri, Japan

## Abstract

This randomized prospective study aimed to evaluate the clinical outcome of denosumab treatment alone and in combination with teriparatide in treatment-naive postmenopausal Japanese female patients with osteoporosis. Thirty patients were randomly assigned to two groups: (1) denosumab group (denosumab alone, *n*=13); and (2) combination group (denosumab+teriparatide, *n*=17). Serum bone-specific alkaline phosphatase (BAP), serum tartrate-resistant acid phosphatase (TRACP)-5b, urinary cross-linked N-terminal telopeptides of type I collagen (NTX), and bone mineral density (BMD) of L1–4 lumbar vertebrae (L-BMD) and bilateral total hips (H-BMD) were determined at the first visit and at various time points up to 24 months post-treatment to determine percentage changes. Serum TRACP-5b and urinary NTX were equally suppressed in both groups and maintained at low levels, with slight increases at 12, 18 and 24 months. BAP was significantly decreased in both groups from 4 to 24 months, with significant differences between the groups at 4, 8 and 15 months (*P*<0.05). L-BMD was significantly increased at most time points in both groups, with a significant difference between the combination group and denosumab group at 24 months (17.2% increase versus 9.6% increase; *P*<0.05). There was no significant difference in H-BMD between the two groups, although the levels tended to be higher in the combination group than in the denosumab group (9.5% increase versus 5.6% increase). These findings suggest that denosumab+teriparatide combination therapy may represent an important treatment for primary osteoporotic patients at high risk of vertebral fracture.

## Introduction

Osteoporosis (OP) is the most common multifactorial metabolic bone disorder worldwide, and is a major public health concern among the elderly and in postmenopausal women. It is therefore of global importance to reduce the burden of this debilitating disease. OP treatments are focused on the prevention of fractures to maintain daily living activities and thereby reduce mortality. Advances in knowledge of bone biology in recent years have contributed to major therapeutic advances in OP treatments.^1^

The fracture rates and proportions of all osteoporotic and hip fractures occurring at various threshold levels of bone mineral density (BMD) were estimated by Siris *et al*.^2^ The estimates indicated that more than 50% of women with hip fractures never completely return to their pre-fracture activities, while 25% are admitted to nursing homes and 20% die within 1 year of fracture. Thus, prevention of hip fractures in elderly patients is critical.

Bisphosphonates (BPs) are the most common drugs administered for OP treatment. With their bone anti-resorptive properties, BPs improve bone turnover and consequently increase both hip and lumbar BMD to prevent hip and vertebral fractures, respectively. Although BPs are the first-line therapy for OP, especially severe OP, teriparatide (TPTD) and/or denosumab may also be administered.^1^

While TPTD remarkably increases lumbar spine BMD,^3,4^ no comparative studies assessing fracture reduction between TPTD and other agents, such as BPs, have been reported to date. Thus, TPTD may be preferentially administered to Japanese OP patients, especially those with greatly diminished BMD and/or multiple vertebral fractures. Although TPTD prevents vertebral fractures and markedly increases L1–4 lumbar vertebrae BMD (L-BMD),^3,5^ there is currently no evidence that TPTD prevents hip fractures. Therefore, TPTD alone may not be suitable for the prevention of hip fractures in elderly patients with OP or those at high risk of hip fractures.

Denosumab is a fully human monoclonal antibody that inhibits receptor activator of nuclear factor kappa-β ligand (RANKL), which selectively inhibits osteoclastogenesis. Recent literature on denosumab showed that the drug progressively and linearly increased BMD in the spine over an 8-year period and increased total hip and femoral neck BMD to a greater extent during the first 3 years of treatment than during years 4–6.^6,7^ Denosumab was also superior in respect to increased BMD and prevented both vertebral and hip fractures.^7^ In elderly OP patients with a high risk of both vertebral and hip fractures, denosumab, or BPs may be appropriate drugs for administration.

As BMD becomes reduced, the frequency of osteoporotic fractures increases. Johnell *et al.*^8^ reported that at the age of 65 years, the risk ratio of femoral neck fracture increases by 1.5–2.0 for each standard deviation (SD) decrease in BMD. In patients with very low BMD, it is therefore essential to ensure that BMD is increased. Thus, in patients with severe OP, combination therapy is expected to be more effective than monotherapy. It remains controversial whether combination treatment with BP and TPTD is superior to monotherapy.^9,10,11,12^

In OP patients, treatment with denosumab increased BMD to a greater extent than treatment with BPs.^12^ Other previous reports showed that combined TPTD and denosumab treatment increased BMD to a greater extent than either agent alone, suggesting that the combination treatment may be useful in patients at high risk of fracture.^6,7^ These findings suggest that combination therapy with denosumab and TPTD may be better than using either agent alone with respect to increasing BMD. However, both treatment-naive and BP-treated patients were included in the above studies.^6,7^

We previously reported that pretreatment with BP before TPTD therapy markedly affected treatment outcomes, such as increased BMD.^4^ To date, no comparative clinical data have been reported on denosumab used alone or in combination with TPTD in treatment-naive primary OP patients. Furthermore, there are no reports in the literature on 2-year treatment with denosumab in Japanese patients with primary OP.

Therefore, we performed a comparative study of changes in bone turnover markers and BMD in 30 Japanese treatment-naive female patients with primary OP. The patients were treated for 2 years with denosumab alone or denosumab combined with TPTD, and examined for their clinical outcomes.

## Materials and methods

### Patient characteristics

The inclusion criteria for the study were primary osteoporotic treatment-naive patients with low L-BMD and/or bilateral hip BMD (H-BMD; less than −3.0 SD). The exclusion criteria were patients with chronic renal failure (estimated glomerular filtration rate <40 mL·min^−1^ per 1.73 m^2^), bone metabolic disorder, or diabetes mellitus, which affect OP, and fracture within 1 year prior to the study. The diagnosis of primary OP was made in accordance with the revised criteria established by the Japanese Society of Bone and Mineral Research.^13^ The study was prospectively performed using simple randomization by an enveloped method.

### Patient classification

**Figure 1:**
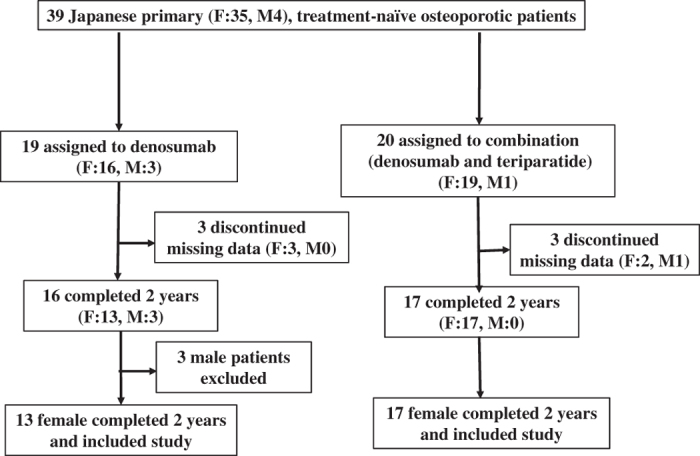
Patient distribution. Of 30 patients, 13 were assigned to the denosumab group and 17 were assigned to the combination treatment (denosumab and teriparatide) group. All patients completed the 2-year visit schedule in this study. F, female; M, male.

The treatment-naive patients with primary OP were classified into two groups: (1) denosumab group, denosumab 60 mg once per 6 months; and (2) combination group, denosumab 60 mg once per 6 months+TPTD 20 μg per day. In total, we collected data for 39 patients who received denosumab (*n*=19) or denosumab+TPTD treatment (*n*=20) between July 2013 and September 2015. Six patients were subsequently excluded from the analysis because of insufficient data collected during the treatment period. As a consequence, only three patients included in the combination group were males. These three male patients were also excluded to avoid gender bias, meaning that only female patients were included in the final analysis. Finally, 13 female patients were included in the denosumab group and 17 female patients were included in the combination group (Figure 1).

In both groups, denosumab 60 mg once per 6 months was administered by injection, and each patient also received vitamin D and calcium supplements (Denotas chewable combination tablets (Daiichi Sankyo, Tokyo, Japan); 762.5 mg of precipitated calcium carbonate, 200 IU of cholecalciferol, and 59.2 mg of magnesium carbonate) twice daily. In the combination group, 20 μg TPTD was subcutaneously injected daily by the patients. Denosumab was injected within 1 week of the start of the TPTD injections. Adherence to TPTD administration and vitamin D and calcium supplementation was assessed based on patient interviews by attending physicians at our institutions.

### Bone turnover markers, whole parathyroid hormone, 1,25(OH)_2_D_3_, and BMD

Serum bone alkaline phosphatase (BAP) was measured as a bone formation marker by a chemiluminescent enzyme immunoassay with inter-assay and intra-assay coefficients of variation (CVs) of 3.0% and 2.5%, respectively. Serum tartrate-resistant acid phosphatase (TRACP)-5b and urinary N-terminal telopeptide of type I collagen (NTX) were assessed as markers of bone resorption. Serum TRACP-5b was measured by an ELISA with inter-assay and intra-assay CVs of 2.2% and 3.2%, respectively. Urinary NTX was measured by an ELISA with inter-assay and intra-assay CVs of 11.5% and 12.7%, respectively. Serum whole parathyroid hormone (PTH 1–84) was measured by an immunoradiometric assay with inter-assay and intra-assay CVs of 2.3% and 2.2%, respectively. Serum active form of vitamin D [1,25(OH)_2_D_3_] was measured by an immunoradiometric assay with inter-assay and intra-assay CVs of 6.0% and 9.5%, respectively. After an overnight fast, serum and first-void urine samples were collected between 08:30 hours and 10:00 hours. Immunoassays were performed by SRL (Tokyo, Japan). Serum samples were stored at −80 °C until bone turnover markers were assessed at the end of the study. Samples were collected before treatment administration, and at 1 week, 1, 2, 4, 8, 12, 15, 18, 21 and 24 months after denosumab or combination treatment.

The percentage changes in bone turnover markers and BMD were determined for each time point, and compared between the groups by statistical analysis. BMD was measured using a dual-energy X-ray absorption (DXA) fan-beam bone densitometer (Lunar Prodigy; GE Healthcare Bio-Sciences, Piscataway, NJ, USA) at the L1–4 levels of the posteroanterior spine and bilateral hips. The CVs of the BMD measurements at the lumbar spine and hip were 0.99% and 0.60%, respectively. The least significant changes in these measurements were 2.7% and 1.6%, respectively. Routine quality control was ensured using a phantom box. Fracture sites were avoided during the evaluation of BMD. H-BMD was calculated as the average BMD of the right and left hips. BMD was examined before treatment administration and at 4, 8, 12, 18 and 24 months. The physicians interpreting the BMD assessments and DXA measurements and the laboratory staff performing the bone marker assays were blinded to the treatment groups.

### Statistical analysis

Results are expressed as mean±standard error of the mean (SE). In both groups, the percentage changes in each marker were determined at each time point (at first administration of denosumab, and at 1 week, 1, 2, 4, 8, 12, 15, 18, 21 and 24 months after first administration) using Holm’s correction method for multiple comparisons. Comparisons of markers between the groups at each time point were performed by Welch’s *t*-test. Based on SD of 2.5% and sample size of 13 in the denosumab group and 17 in the combination group, we calculated that the study had 80% power to detect at least a 5% difference in lumbar BMD. Significant differences were considered when *P*<0.05.

This study was approved by the institutional ethics committee of Shinshu University School of Medicine and Showa Inan General Hospital, and informed consent was obtained from all patients. The methods were carried out in accordance with approved guidelines. The clinical trial registration number is NCT02156960, and the date of registration was 31 May 2014.

## Results

All 30 enrolled female patients completed the observational visits over the 2-year study period. There were no significant differences between the groups in patient age, BMI, H-BMD, or L-BMD before treatment (Table 1). All patients in the combination group reported taking over 95% of TPTD doses. All patients in both groups received all expected denosumab doses every 6 months. Serious adverse events, including hypocalcemia or fracture, were not reported during the study.

### Serum calcium corrected by albumin and phosphorus levels

**Figure 2:**
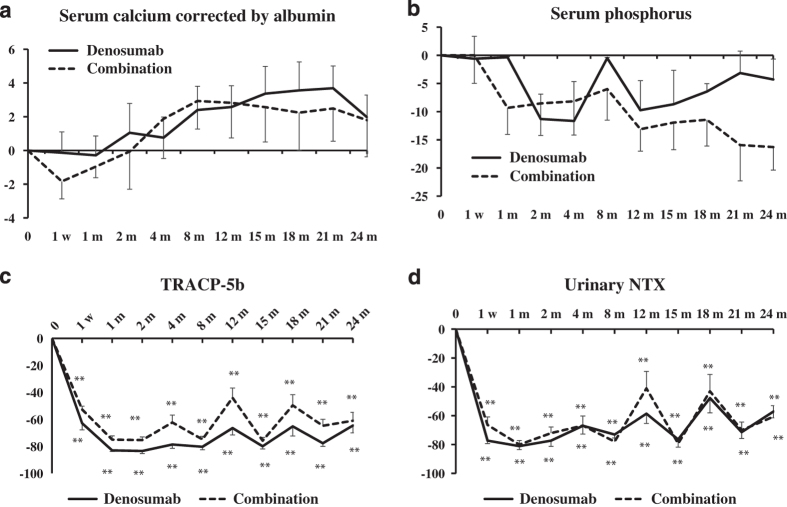
Percentage changes in serum albumin-corrected calcium (**a**), serum phosphorus (**b**), serum tartrate-resistant acid phosphatase (TRACP)-5b (**c**), and urinary N-terminal telopeptide of type I collagen (NTX) (**d**) at pretreatment and at 1 week (w), 1, 2, 4, 8, 12, 15, 18, 21 and 24 months (m). Solid line: denosumab group; dotted line: combination (denosumab and teriparatide) group. Results are expressed as mean±s.e. ^#^*P*<0.05, significant difference between the denosumab and combination groups at each time point. **P<0.01, significant difference at 1 week (w), 1, 2, 4, 8, 12, 15, 18, 21 and 24 months (m), compared with pretreatment in either the denosumab or combination group.

The percentage changes in albumin-corrected serum calcium (Ca) after treatment did not differ significantly between the groups during the observation period. All Ca levels were within the normal range (Figure 2a). The percentage changes in serum phosphorus after treatment showed no significant differences between the groups or between individual time points within each group, compared with the values before treatment. All phosphorus levels were within the normal range (Figure 2b).

### Bone turnover markers

With respect to bone resorption markers, the percentage changes in serum TRACP-5b were significantly decreased at each time point in both groups compared with the pretreatment values. There were no significant differences between the groups, although the percentage changes in TRACP-5b tended to be higher in the combination group than those in the denosumab group. In both groups, the decreased percentage changes in TRACP-5b increased again at 12, 18 and 24 months (Figure 2c).

Similarly, the percentage changes in urinary NTX were significantly decreased at each time point in both groups, except for 18 months in the combination group, compared with the pretreatment values. There were no significant differences in the percentage changes in urinary NTX at each time point between the groups. Similar to TRACP-5b, the decreased percent changes of urinary NTX increased again at 12, 18 and 24 months (Figure 2d).

**Figure 3:**
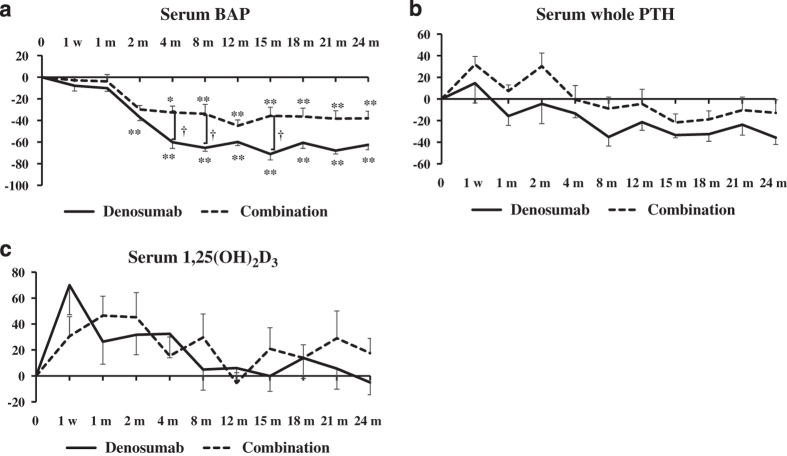
Percentage changes in serum bone alkaline phosphatase (BAP) (**a**), serum whole parathyroid hormone (PTH) (**b**), and serum 1,25(OH)_2_D_3_ (**c**) at pretreatment and at 1 week (w), 1, 2, 4, 8, 12, 15, 18, 21 and 24 months (m). Solid line: denosumab group; dotted line: combination (denosumab and teriparatide) group. Results are expressed as mean±s.e. ^†^*P*<0.05, significant difference between the denosumab and combination groups at each time point. **P<0.01, significant difference at 1 week (w), 1, 2, 4, 8, 12, 15, 18, 21 and 24 months (m) compared with pretreatment in either the denosumab or combination group.

With respect to bone formation markers, the percentage changes in BAP were significantly decreased between 2 and 24 months in the denosumab group and between 4 and 24 months in the combination group, compared with the pretreatment values. The percentage changes in BAP in the denosumab group were significantly decreased at 4, 8, and 15 months compared with the combination group (*P*<0.05; Figure 3a).

### Serum whole PTH and 1,25(OH)_2_D_3_

The percentage changes in whole PTH tended to be lower in the denosumab group than in the combination group. There were no significant differences between the groups (Figure 3b).

There were no significant differences in the percentage changes in serum 1,25(OH)_2_D_3_ in both groups at all time points or between the two groups (Figure 3c).

### L-BMD and H-BMD

**Figure 4:**
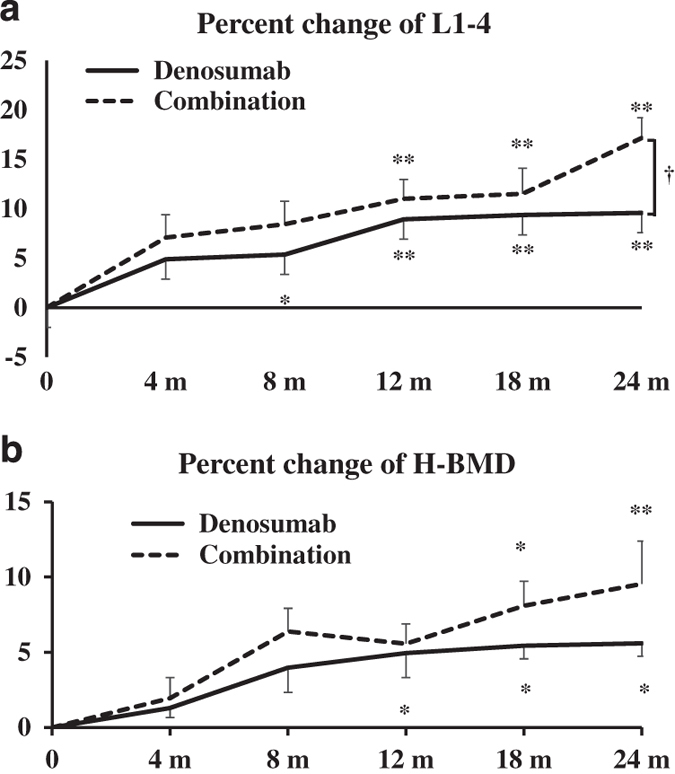
Percentage changes in bone mineral density (BMD) of L1–4 lumbar vertebrae (L-BMD) (**a**) and bilateral total hips (H-BMD) (**b**) at the first visit and at 4, 8, 12, 18 and 24 months. Results are expressed as mean±s.e. ^†^*P*<0.05, significant difference between the denosumab and combination groups at each time point. *P<0.05, **P<0.01, significant difference at 4, 8, 12, 15, 18 and 24 months compared with pretreatment in either the denosumab or combination group.

The percentage changes in L-BMD increased steadily from pretreatment to 24 months post-treatment in the denosumab group (9.6% increase) and to a greater extent in the combination group (17.2% increase; Figure 4a). The percentage changes in L-BMD were significantly increased in both groups at each time point, except at 4 months in the denosumab group, and at 4 and 8 months in the combination group, compared with the pretreatment levels. There was a significant difference between the groups at 24 months (*P*<0.05; Figure 4a).

The percentage changes in H-BMD increased steadily from pretreatment to 24 months post-treatment in the denosumab group (5.6% increase) and in the combination group (9.5% increase). The values were significantly increased in the combination group at 18 and 24 months and in the denosumab group at 12, 18 and 24 months, compared with the pretreatment values. Although the percentage changes in the combination group were greater than those in the denosumab group, there were no significant differences between the groups (Figure 4b).

## Discussion

The present study examined the percentage changes in BMD and bone turnover markers in 30 Japanese treatment-naive postmenopausal female patients with primary OP treated with denosumab alone or in combination with TPTD. In the denosumab group, L-BMD and H-BMD increased from pretreatment to 24 months by 9.6% and 5.6%, respectively, and in the combination group by 17.2% and 9.5%, respectively. These findings suggest that the combination therapy may be of value for the treatment of women with OP and a high risk of bone fragility fractures. This is the first study to report comparative data on denosumab combination therapy versus denosumab alone in this type of patient population.

TPTD is a bone-forming drug that acts by accelerating bone metabolism. When administered in the presence of anti-resorptive drugs, such as BPs, there are concerns regarding the inhibition of its effects. Interestingly, PTH administration after long-term BP treatment accelerated bone turnover markers, although BMD was compromised.^4^ These findings suggest that denosumab, which strongly inhibits bone metabolism, might negatively affect bone metabolism when combined with TPTD. However, Tsai *et al.*^6^ reported that combination therapy of TPTD with denosumab increased BMD to a greater extent than TPTD monotherapy. Thus, the mechanisms by which TPTD therapy in combination with anti-resorptive drugs act on bone metabolism in OP remain unknown.

Our findings are comparable to those from previous studies.^6,7^ Leder *et al.*^7^ reported that BMD changes in the second year of therapy were generally similar across all treatment groups at all anatomical sites, and that the most cost-effective way to achieve greater increases in BMD may be to administer combined TPTD and denosumab treatment for 1 year followed by anti-resorptive agent treatment alone for the second year. Patients who had taken BPs within 6 months before enrollment were excluded from their study, while patients who had taken BPs earlier than 6 months prior to enrollment were included. Nevertheless, Leder *et al.*^7^ also demonstrated the superiority of the combination therapy in women with or without prior BP exposure. The treatment effects of TPTD with respect to BP treatment prior to TPTD therapy may be race-dependent.^4,14^ Obermayer-Pietsch *et al.*^14^ reported that the effects of TPTD on BMD were moderately decreased by pretreatment with BP. However, our previous study showed that BP pretreatment significantly reduced the increases in BMD observed following TPTD treatment.^4^ Meanwhile, BMD values can be increased in BP-unresponsive patients following denosumab treatment.^15^ The terminal elimination half-life of BPs, even in rats, is considerably more than 1 year. For example, the half-life of alendronate was ~200 days (0.55 years) in the femur, that of ibandronate was 500 days (1.5 years) in the femoral metaphysis, and that of minodronate was 451 days (1.24 years) in the humerus.^16,17^ Thus, in Japanese patients, pretreatment with BP for more than 6 months may affect BMD levels following TPTD and denosumab therapy. Thus, patients with OP pretreatment history were excluded from the present study.

Generally, bone resorption and bone formation change in parallel through the phenomenon of coupling.^18^ Tsai *et al.*^6^ previously reported that in the combination group, bone formation was not increased, but rather decreased less than that observed with monotherapy, while the percentage changes in a resorption marker (cross-linked C-terminal telopeptide of type I collagen) were the same in the denosumab monotherapy and combination groups. In the present study, denosumab alone and in combination resulted in strong bone resorption from the early stages of treatment with no clearly evident inhibitory effects on bone formation markers, comparable to the findings of our previous study and other studies.^6,7,19^ The combined therapy decreased bone formation (as assessed by BAP) to a lesser extent than denosumab monotherapy, which may have contributed to the larger BMD increase.

The mechanism by which the denosumab and TPTD combination therapy increases BMD, while BP and TPTD combination therapy does not, remains unknown. Gatti *et al.*^20^ reported on the different anti-OP mechanisms of denosumab and BP activity via Wnt signaling. It was previously reported that canonical Wnt signaling decreases bone resorption.^18^ Gatti *et al.*^20^ concluded that denosumab combined with long-term BP treatment increased sclerostin, which antagonizes canonical Wnt signaling, and that denosumab decreased Dkk1, another major canonical Wnt antagonist.^21^ However, BP did not affect Wnt signaling; therefore, these points might explain why denosumab and BP use different mechanisms to regulate bone metabolism (for example, BMD), especially in combination with TPTD.^20,22^

The limitations of this study include the small sample size, short follow-up period, and lack of evaluation of fracture prevention during the study.

In conclusion, combination therapy of denosumab and TPTD remarkably increased L-BMD and H-BMD compared with denosumab alone. Thus, this combination therapy could be of benefit for the treatment of Japanese primary OP patients with a high risk of vertebral and hip fractures.

## Figures and Tables

**Table 1:** Patient characteristics

Characteristic	Denosumab (*n*=13)	Combination (*n*=17)	*P*-value
Age/years	75.1±1.8	75.5±1.4	*P*=0.841 3
BMI/(kg·m^−2^)	20.9±0.9	21.4±1.0	*P*=0.717 1
Serum corrected Ca/(mg·dL^−1^)	9.0±0.1	9.0±0.1	*P*=0.917 5
Serum phosphorus/(mg·dL^−1^)	3.8±0.1	3.7±0.1	*P*=0.825 4
Serum BAP/(μg·L^−1^)	21.6±2.5	19.2±2.4	*P*=0.497 6
Serum TRACP-5b/(mU·dL^−1^)	587.5±33.5	571.5±48.8	*P*=0.788 6
Urinary NTX (nmol BCE per mmol CRE)	60.4±9.1	66.0±8.0	*P*=0.644 0
1, 25 (OH)_2_D_3_/(pg·mL^−1^)	56.1±5.6	58.2±4.3	*P*=0.773 4
Serum whole PTH/(pg·mL^−1^)	35.7±2.8	28.7±3.4	*P*=0.126 7
L1–4 BMD/(g·cm^−2^)	0.799±0.03	0.730±0.03	*P*=0.153 0
Total hip BMD/(g·cm^−2^)	0.64±0.03	0.62±0.01	*P*=0.456 3

BAP, bone-specific alkaline phosphatase; BMD, bone mineral density; BMI, body mass index; NTX, N-terminal telopeptides of type I collagen; PTH, parathyroid hormone; TRACP, tartrate-resistant acid phosphatase.

Results are expressed as mean±s.e.

Differences of *P*<0.05 were considered significant.
